# The PI3K/Akt pathway: a critical player in intervertebral disc degeneration

**DOI:** 10.18632/oncotarget.18628

**Published:** 2017-06-27

**Authors:** Zhi-Hua Ouyang, Wen-Jun Wang, Yi-Guo Yan, Bing Wang, Guo-Hua Lv

**Affiliations:** ^1^ Department of Spine Surgery, The 2nd Xiangya Hospital of Central South University, Changsha, China; ^2^ Department of Spine Surgery, The First Affiliated Hospital, University of South China, Hengyang, China

**Keywords:** IDD, PI3K, Akt, PTEN, mTOR

## Abstract

Intervertebral disc degeneration (IDD) is thought to be the primary cause of low back pain, a severe public health problem worldwide. Current therapy for IDD aims to alleviate the symptoms and does not target the underlying pathological alternations within the disc. Activation of the phosphatidylinositol 3-kinase (PI3K)/Akt pathway protects against IDD, which is attributed to increase of ECM content, prevention of cell apoptosis, facilitation of cell proliferation, induction or prevention of cell autophagy, alleviation of oxidative damage, and adaptation of hypoxic microenvironment. In the current review, we summarize recent progression on activation and negative regulation of the PI3K/Akt signaling pathway, and highlight its impact on IDD. Targeting this pathway could become an attractive therapeutic strategy for IDD in the near future.

## INTRODUCTION

Low back pain (LBP) is a major public health problem worldwide, resulting in a huge socioeconomic burden [1–4]. Although the etiology of LBP is complex, intervertebral disc degeneration (IDD) has been regarded as the primary cause [5]. It is acknowledged that genetic predisposition, aging and lifestyles including occupational exposure, smoking, and alcohol consumption are implicated in disc degeneration [6–9]. Although its pathogenesis is not fully elucidated, loss of active IVD cells, progressive breakdown of extracellular matrix (ECM), alternation of intervertebral disc (IVD) cell phenotypes, and excessive inflammatory response have been proposed as critical contributors to IDD [10, 11]. Current therapy for IDD aims to relieve pain and control symptom rather than interfere with its pathophysiology [12]. Although several biological strategies capable of slowing mild and moderate disc degeneration through induction of ECM remodeling have been considered [13–17], their clinical efficacy remains to be determined. Therefore, a better understanding of its pathogenesis is essential to develop effective treatments.

The intervertebral disc (IVD) is made up of the central nucleus pulposus (NP), the peripheral annulus fibrosus (AF) enclosing NP, and the upper and lower cartilaginous endplates (CEPs) [18]. These three distinct anatomic regions form a complicated structure for maintaining spinal stability. The IVD is the biggest avascular tissue in human body, with limited blood supply via peripheral capillaries [19]. Given the fact that the IVD resides in a nutrient deficient environment, disc degeneration is prone to occur if nutrition is depleted or impeded.

Dysregulation of multiple signaling pathways has been shown to be implicated in disc degeneration [20]. As a main intracellular signaling pathways, activation of the phosphatidylinositol 3-kinase (PI3K)/Akt pathway can modulate cell proliferation, apoptosis, autophagy and differentiation under the physiological and pathological conditions by interacting with multiple downstream target proteins, including mammalian target of rapamycin (mTOR) and forkhead box O1 (FoxO1) [21–23]. More importantly, targeting this pathway has shown promise for the prevention or reversal of IDD. In this review, after introduction of the PI3K/Akt pathway, we explore its role in disc degeneration.

## The PI3K/AKT SIGNALING PATHWAY AND NEGATIVE REGULATORS

### Activation of the PI3K/Akt signaling pathway and downstream effectors

As a unique family of intracellular lipid kinases, PI3Ks contain three classes (Class I, II and III). Among these, Class I is the most studied, which is subdivided into class IA (PI3Kα, β and δ) and class IB (PI3Kγ). Class I PI3Ks are heterodimer that are made up of a catalytic subunit (p110α, β, γ or δ,) and a regulatory subunit (p85α, β or γ) [24]. Akt, a serine/threonine kinase, includes three isoforms (Akt1, 2, and 3). These isoforms have high homology [25].

**Figure 1:**
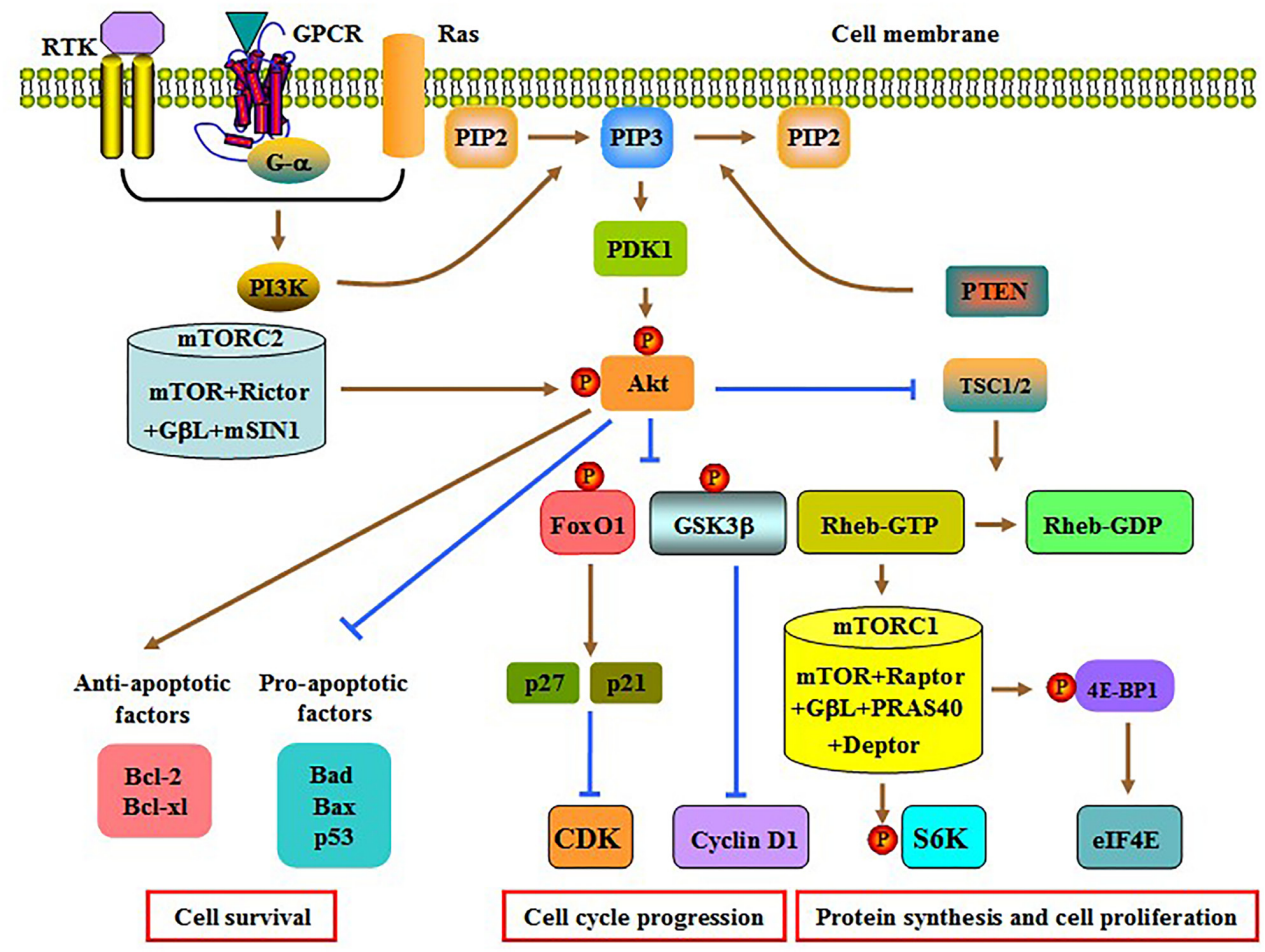
Schematic diagram of the PI3K/AKT pathway PI3K is activated upon binding of an extracellular ligand to RTK, GPCR or Ras. Activated PI3K converts PIP2 to PIP3, which is able to be reversed by PTEN. PIP3 then recruits PDK1, which phosphorylates and partially activates Akt. The mTORC2 mediates the second phosphorylation to fully activate AKT. Subsequently, the formation of the TSC1/2 heterodimer is significantly decreased, which leads to a marked increase in Rheb-GTP content and mTORC1 activation. The mTORC1 induces S6K phosphorylation to release 4E-BP1, which stimulate protein synthesis and cell proliferation. Akt activation enhances cyclin D1 and CDK expression by inhibiting GSK3β and FoxO1, respectively. Both effects contribute to cell cycle progression. Activated Akt can also suppress apoptosis via regulating the expression of apoptosis-associated genes. Raptor, regulatory-associated protein of mTOR; GβL, mammalian LST8/G-protein β-subunit like protein; PRAS40, proline-rich Akt substrate 40; Deptor, DEP domain containing mTOR-interacting protein; mSIN1, mammalian stress-activated protein kinase interacting protein 1.

Class IA and class IB PI3Ks are activated by different agents. Receptor tyrosine kinases (RTKs) are transmembrane glycoproteins with enzymatic activity. PI3Kα, PI3Kβ and PI3Kδ are activated when an extracellular ligand binds to a RTK [26]. In contrast, both G-protein-coupled receptors (GPCRs) and small GTPase Ras are responsible for PI3Kγ activation [22]. Once activated, PI3Ks convert membrane-bound phosphatidylinositol 4,5-biphosphate (PIP2) into phosphatidylinositol 3,4,5-triphosphate (PIP3) [27]. PIP3 then recruits phosphoinositide- dependent kinase 1 (PDK1) to phosphorylate Akt at threonine 308 (Thr308). Subsequently, mTOR complex 2 (mTORC2) phosphorylates Akt at serine 473 (Ser473) for full AKT activation [28, 29]. Thereafter, activated Akt interacts with downstream target proteins to regulate multiple biological processes, including apoptosis, autophagy and cell cycle progression (Figure 1).

As an evolutionarily conserved serine/threonine protein kinase from yeast to mankind, mTOR interacts with other proteins to form two multiprotein complexes: mTORC1 and mTORC2 [30]. The mTORC1 is extremely sensitive to rapamycin, whereas mTORC2 is insensitive to rapamycin. The activated Akt reduces the formation of tuberous sclerosis complex (TSC) 1/2 and then inhibits the conversion of GTP-Rheb to GDP-Rheb. Increased GTP-Rheb activates mTORC1 [31]. Subsequently, mTORC1 induces the phosphorylation of ribosomal protein S6 kinase (S6K) and eukaryotic translation initiation factor 4E-binding protein 1 (4E-BP1). Once phosphorylated, the latter stimulates the release of eukaryotic translation initiation factor 4E (eIF4E). Both S6K and eIF4E finally promote protein translation and cell proliferation [32, 33].

Besides mTORC1, Akt is capable of deactivating both glycogen synthase kinase 3β (GSK3β) and FoxO1 by stimulating their phosphorylation [34, 35]. Inactivation of GSK3β increases the biogenesis of cyclin D1 and then accelerates cell cycle. FoxO1 contains a conserved forkhead domain and three putative phosphorylation sites for Akt. Decreased activity of FoxO1 restrains the transcription of p27 and p21, two inhibitors of cyclin-dependent kinase (CDK), resulting in cell cycle progression [36]. Activated PI3K/Akt signaling can also facilitate cell survival via attenuating pro-apoptotic Bad, Bax and p53 levels as well as enhancing anti-apoptotic Bcl-2 and Bcl-xl levels [37, 38].

#### Negative regulators

The major negative regulator of this pathway is phosphatase and tensin homolog (PTEN) that converts PIP3 back to PIP2, an opposite action with PI3K (Figure 1) [39, 40]. Notably, the activated PI3K/Akt pathway can also facilitate ubiquitin-mediated proteasomal cleavage of PTEN by upregulating the expression of NEDD4-1 (an E3 ligase), leading to permanent activation of this pathway in a positive feedback loop [41, 42]. In addition to PTEN, there are some specific inhibitors targeting PI3K or Akt. The PI3K inhibitors primarily contain LY294002, BYL719 and BKM120. The Akt inhibitors consist of MK-2206, GSK690693 and RX0201 [43].

## ROLES OF THE PI3K/AKT PATHWAY IN THE PATHOGENESIS OF IDD

**Figure 2:**
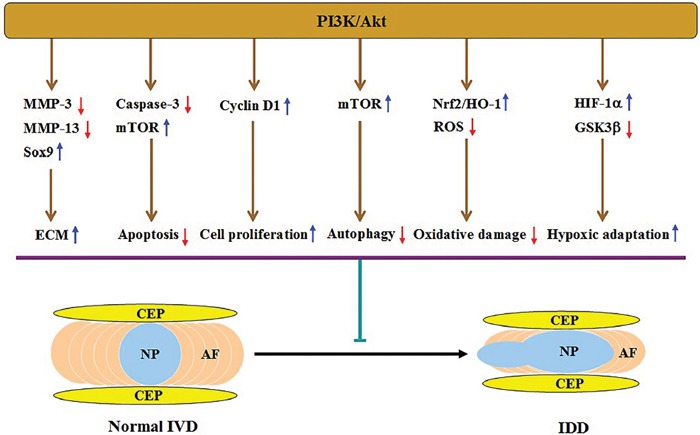
Illustration of PI3K/Akt-mediated protection against IDD and the underlying mechanisms Activation of this pathway increases ECM content via downregulating MMP-3 and MMP-13 expression and upregulating Sox9 expression, inhibits apoptosis by activating mTOR and attenuating caspase-3 activity, and promotes cell proliferation by upregulating cyclin D1 expression. This pathway can also suppress autophagy by activating mTOR, alleviate oxidative injury via activating the Nrf2/HO-1 signaling and decreasing ROS levels, and enhance adaptation of hypoxic microenvironment by upregulating HIF-1α expression and inactivating GSK3β. All of these effects result in alleviation of disc degeneration.

IDD is a complicated disease involving numerous pathologic processes [44]. As expected, the PI3K/Akt pathway takes part in disc degeneration through multiple mechanisms (Figure 2), which will be discussed in detail below.

### Increase of ECM content

The hallmark of IDD is progressive loss of ECM components due to increased production of matrix metalloproteinases (MMPs) and a disintegrin and metalloprotease with thrombospondin motifs (ADAMTSs) [45, 46]. Insulin-like growth factor-1 (IGF-1) has a significant protective effect against IDD [47–49]. Serum (IGF-1) levels were lower in lumbar disc degeneration patients than those of healthy controls, and addition of IGF-1 to human NP SV40 cells induced Akt phosphorylation and then inactivated FoxO1, leading to inhibition of MMP-3 transcription [50]. In rat endplate chondrocytes, addition of IGF-1 dramatically decreased the expression and activity of MMP-13 by activating the PI3K/Akt pathway, which increased type II collagen (Col II) content [51]. Additionally, treatment of bovine NP cells with IGF-1 combined bone morphogenetic protein 7 (BMP-7) synergistically promoted aggrecan accumulation by enhancing Akt activity [52]. Accumulating evidence suggests that dysregulation of microRNAs (miRNAs) participates in disc degeneration [53, 54]. Recently, Liu and colleagues observed that miR-4458 levels were significantly increased in degenerative human lumbar disc specimens [55]. They also found that transfection of human NP cells with miR-4458 mimic inhibited the PI3K/Akt signaling via silencing of insulin-like growth factor 1 receptor (IGF-1R), leading to increased ECM breakdown [55]. Collectively, activated PI3K/Akt pathway contributes to prevention of disc ECM degradation.

In addition to blockade of ECM catabolism, activation of this pathway promotes its anabolism. Sox9 is an important transcriptional factor that can drive *aggrecan* gene expression [56–58]. It has been reported that miR-30a attenuates aggrecan content by targeting Sox9 in primary chondrocytes from cartilage isolated from osteoarthritis donors [59]. In contrast, overexpression of Sox9 dramatically facilitated aggrecan production in cultured human articular chondrocytes [60]. Similarly, activation of the PI3K/AKT signaling increased Sox9 expression and activity, which subsequently induced transcription of *aggrecan* gene in rat NP cells [61].

### Inhibition of cell apoptosis

In general, apoptosis occurs via two well-characterized pathways in mammalian cells: the death receptor or extrinsic pathway and mitochondria or intrinsic pathway [62]. IVD cell loss resulting from excessive apoptosis has long been considered to be an important cause of reduced ECM synthesis during disc degeneration [63–65]. In addition to modulation of intradiscal ECM metabolism, this pathway can suppress apoptosis of IVD cells. Administration of 17β-estradiol combined with resveratrol (a natural polyphenol compound) promoted Akt phosphorylation and decreased caspase-3 activity, leading to apoptosis inhibition in rat NP cells treated with interleukin-1β (IL-1β) [66]. Transforming growth factor-β1 (TGF-β1) is a key factor during the development of both cartilage and spine tissues [67, 68]. Mesenchymal stem cell transplantation with pure fibrinous gelatin-TGF-β1 markedly decreased degenerative degree in a rabbit IDD model [69]. Treatment of rat AF cells with TGF-β1 was found to reduce apoptosis incidence by activating the PI3K/AKT/mTOR pathway under serum deprivation [70]. Conversely, miR-27a overexpression induced apoptosis of human degenerated NP cells via silencing of PI3K [71]. Thus, *in vivo* delivery of miR-27a inhibitors might be a promising therapeutic strategy to restore the number of viable NP cells for IDD patients.

Sirtuin 1 (SIRT1) is a NAD+-dependent class III histone deacetylase [72]. The protective effects of SIRT1 against disc degeneration are primarily derived from its abilities to promote ECM anabolism, inhibit inflammatory response and alleviate senescence of CEP cells [73–76]. It has been demonstrated that SIRT1 markedly decreases the rate of apoptosis in multiple cell types, such as osteoblast-like MC3T3-E1 cells [77], human kidney proximal tubule epithelial cells [78], cardiomyocytes [79], and chondrocytes [80]. In human degenerative NP cells, SIRT1 was also reported to protect against apoptosis via autophagic induction [81, 82]. Notably, resveratrol-induced SIRT1 activation stimulated Akt phosphorylation and reduced apoptotic incidence of human NP cells, whereas either Akt knockdown or LY294002 abrogated the inhibitory effect of SIRT1 on NP cell apoptosis [83]. In a later study, miR-138-5p knockdown upregulated the expression of its target gene SIRT1 and then inhibited apoptosis in human NP cells treated by tumor necrosis factor-α (TNF-α) [84]. Mechanistically, upregulation of SIRT1 decreased PTEN levels to activate the PI3K/Akt pathway [84]. These findings reveal the PI3K/AKT signaling as another important mechanism for SIRT1-mediated inhibition of NP cell apoptosis. Targeting this pathway might have enormous potential for retarding or reversing disc degeneration.

### Promotion of cell proliferation

Appropriate proliferation of IVD cells represents a tissue repair process during disc degeneration [85]. In addition to blockade of IVD cell apoptosis, this pathway can raise the number of viable IVD cells by promoting cell proliferation. In human IVD cells, exogenous treatment with either PDGF or IGF-1 stimulated DNA synthesis, which was at least partially attributed to activation of this pathway [86]. A similar effect was also observed in bovine coccygeal NP and AF cells [87]. Conversely, high osmolality significantly reduced Akt phosphorylation and then inhibited PDGF or IGF-I-induced synthesis of novel DNA in bovine NP cells [88]. Leptin belongs to a peptide hormone, and its plasma levels are significantly increased in obesity patients. It can be produced by fibrocartilaginous tissues, such as articular cartilage and IVD, besides adipose tissues [89]. Recently, Zhao et al. reported that human herniated disc tissues expressed leptin and its functional receptor, and administration of leptin could stimulate rat NP cell proliferation [90]. Leptin also promoted the proliferation of primary cultured human NP cells [91]. Mechanistically, leptin increased cyclin D1 expression via inhibition of Akt phosphorylation [91]. Thus, the PI3K/AKT signaling may function as a cross-talk between obesity and IDD.

MiR-21 functions as an inducer of cell proliferation [92–94]. Interestingly, transfection of human NP cells with miR-21 mimic dramatically increased Akt activity via targeting PTEN, leading to upregulation of cyclin D1 expression and subsequent cell proliferation [95]. It is worth noting that overexpression of miR-21 could also stimulate these cell proliferation via silencing of programmed cell death 4 (PDCD4) [96]. Recently, Li et al. identified growth arrest specific gene 1 (GAS1) as a direct and functional target of miR-184 [97]. Moreover, ectopic expression of miR-184 markedly attenuated GAS1 levels, which promoted Akt phosphorylation and human NP cell proliferation [97]. Like miR-21 and miR-184, miR-10b is also a multi-functional miRNA. Aberrant expression of this miRNA contributed to the proliferation of malignant tumor cells [98–100]. Similar to these reports, transfection of human NP cells downregulated homeobox D10 (HOXD10) expression, leading to increased Akt phosphorylation and cell proliferation [101]. However, restored expression of HOXD10 or Akt suppression reversed the mitogenic effect of miR-10b [101]. Taken together, miR-21, miR-184 and miR-10b promote NP cell proliferation by activating the PI3K/AKT signaling.

### Regulation of cell autophagy

Autophagy is an orchestrated homeostatic process involving the degradation and digestion of intracellular components by lysosomes [102, 103]. Recent studies have focused on the relationship between autophagy and IDD [104]. There is a low basal level of autophagy in normal NP and AF cells [105]. In contrast, degenerative NP and AF cells exhibited a significant increase in the autophagic activity [106, 107]. When compared with healthy controls, human degenerated AF tissues showed more autophagic vacuolization and autophagosomes [108]. In NP cells isolated from rat lumbar discs, hydrogen peroxide (H_2_O_2_) induced an early autophagic response via the extracellular signal-regulated kinase (ERK)/mTOR pathway; however, prevention of autophagy by 3-methyladenine (3-MA) markedly attenuated the apoptosis of H_2_O_2_-treated NP cells [109]. On the other hand, human degenerative NP cells exhibited a marked decrease in autophagy activity [81]. Importantly, activation of autophagy by rapamycin suppressed IL-1β-induced ECM degradation in rat NP cells [110]. Thus, autophagy acts as a double-edged sword in the development of IDD, depending on the stimuli.

**Figure 3:**
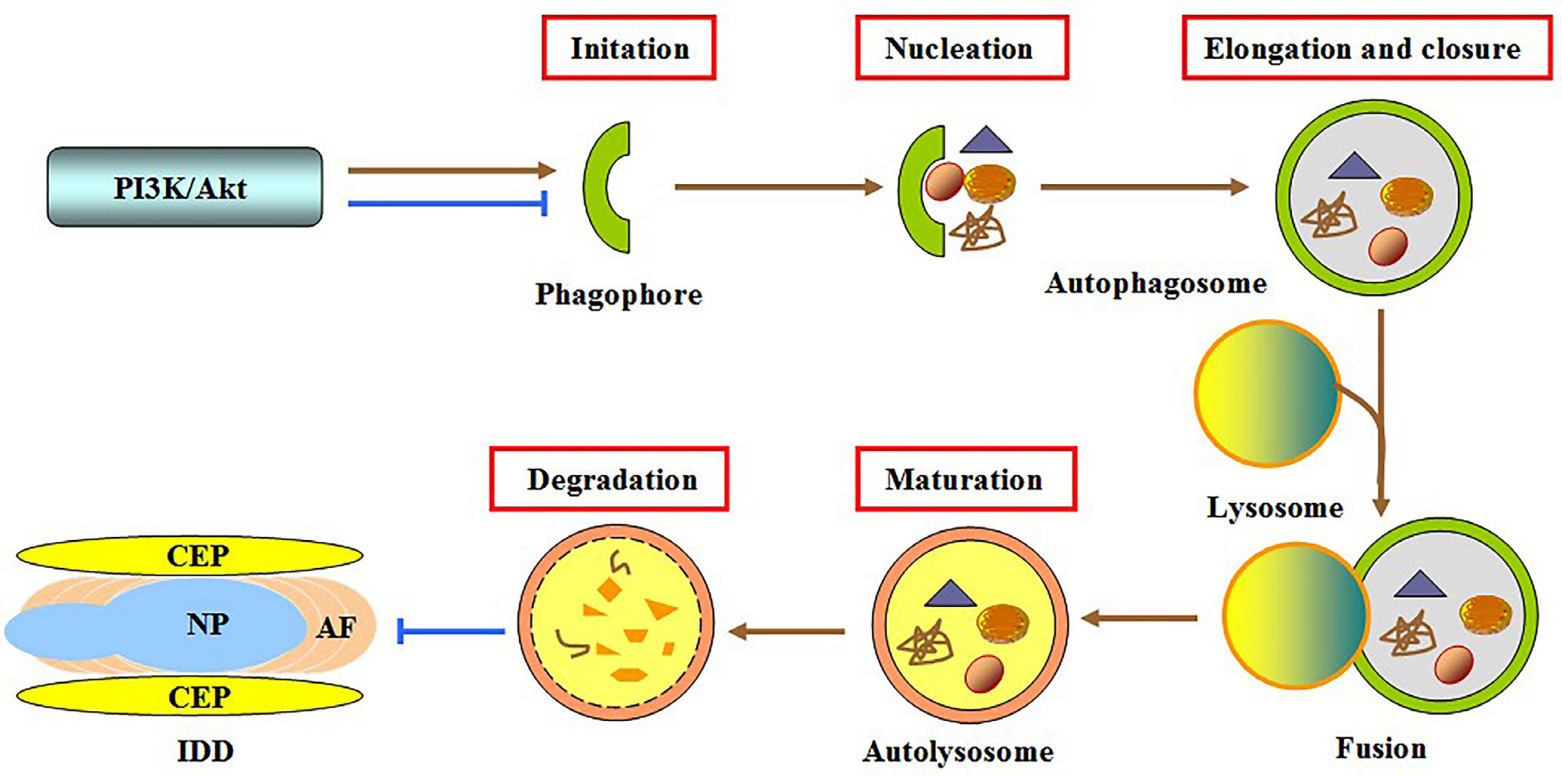
Involvement of autophagy in PI3K/Akt-mediated protection against IDD Autophagy is a successive process involving the formation of phagophores, autophagosomes and autolysosomes, and degradation of vesicle contents. Activating the PI3K/AKT pathway can antagonize or induce IVD cell autophagy, thereby leading to inhibition of disc degeneration.

The PI3K/Akt pathway is closely associated with autophagy [111, 112]. Recently, Ni et reported that treatment of rat AF cells with TGF-β1 markedly decreased autophagy incidence by activating the PI3K/AKT/mTOR signaling pathway under serum deprivation, leading to increased viable cell number [70]. Notably, IGF1 promoted the survival of human NP cells exposed to compression via autophagy induction, which was abrogated by the specific Akt inhibitor LY294002 [113]. These findings reveal a complex role of this pathway in regulating IVD cell autophagy. This discrepancy may be attributed to the differences in stimuli, cell types and downstream effectors. However, both alterations of autophagy activity contribute to its protection against IDD (Figure 3).

### Alleviation of oxidative damage

Oxidative stress is a critical contributor to disc degeneration, because it not only reinforces ECM degradation and inflammatory response, but also reduces the viability and number of IVD cells [114, 115]. Once activated, the PI3K/Akt pathway can enhance endogenous antioxidant ability in multiple cell types, such as macrophages [116], human umbilical vascular endothelial cells [117], and intestinal epithelial cells [117]. Epigallocatechin 3-gallate (EGCG) is known to delay painful disc degeneration by reducing inflammation and catabolism [118]. Recently, Krupkova et al. reported that EGCG administration protected human degenerative NP cells from oxidative stress by promoting Akt phosphorylation [118]. This reveals a novel mechanism by which EGCG protects against IDD. It has been believed that activation of nuclear factor erythroid 2-related factor 2 (Nrf2)/heme oxygenase-1 (HO-1) signaling pathway stimulates the synthesis of antioxidant enzymes such as superoxide dismutase (SOD), catalase (CAT) and glutathione-peroxidase (GSH-Px), leading to enhancement of antioxidant defense system [119, 120]. Moreover, activated PI3K/Akt signaling can promote nuclear translocation of Nrf2 and subsequent upregulation of HO-1 expression under oxidative stress [121, 122]. Thus, it is likely that activation of Nrf2/HO-1 pathway by the PI3K/Akt signaling is, at least partially, responsible for EGCG-mediated attenuation of oxidative damage during IDD.

Mitochondrion dysfunction is a major cause of excessive reactive oxygen species (ROS) production, the hallmark of oxidative stress. ROS has been shown to induce NP and AF cell apoptosis through the mitochondria pathway [109, 123, 124]. Noticeably, the PI3K/Akt signaling pathway can affect ROS-stimulated apoptosis in these cells. For example, administration of chlorogenic acid markedly decreased ROS production and inhibited apoptosis by activating the PI3K/Akt signaling in H_2_O_2_-treated rat NP cells [125]. In human degenerative NP cells exposed to H2O2, EGCG promoted the phosphorylation of Akt, leading to decreased mitochondrial membrane depolarization and increased cellular survival [118]. Taking these findings into account, activating this signaling cascade may be an important approach for alleviation of intradiscal oxidative damage by blocking the mitochondria apoptotic pathway.

### Adaptation of hypoxic microenvironment

Since the disc is avascular, the main components within the disc, including water, aggrecan and fibrillar collagens, proportionally vary considerably depending on the location across the disc. Moreover, there is always a steep concentration gradient of oxygen across the disc, with pO2 falling to as low as 1 % in the center of the disc. Therefore, the IVD resides in a hypoxic microenvironment, which is essential for maintaining its normal physiological functions, including cellular metabolism and protein synthesis [126, 127].

The IVD cells have developed some important mechanisms to ensure their survival in the hypoxic microenvironment of the disc. Hypoxia-inducible factor-1α (HIF-1α), an important transcription factor, is responsible for the induction of genes that facilitates adaptation and survival of cells and tissues under hypoxia condition [128]. Risbud et al. detected HIF-1α expression in rat, human, and sheep NP cells in the presence of either hypoxia or normoxia, and found that these cells consistently expressed functionally active HIF-1α in the presence of hypoxia [129]. Given the fact that activation of the PI3K/Akt signaling can upregulate HIF-1α expression under hypoxia in multiple cell types [130, 131], this pathway may be also implicated in upregulation of HIF-1α expression induced by hypoxia in NP cells. On the other hand, Risbud et al. observed that under serum starvation condition, hypoxic contributed to attenuating apoptosis of rat NP cells [132]. Moreover, cell survival in response to hypoxia was associated with activation of the PI3K/Akt/GSK3β signaling pathway [133]. Thus, activated PI3K/Akt pathway can enhance the adaptation of NP cells to hypoxic microenvironment.

## CONCLUSIONS AND FUTURE PERSPECTIVES

Although activated PI3K/Akt pathway has been shown to protect against disc degeneration through multiple mechanisms, there are a number of outstanding issues that need to be addressed. One of the most important aspects is the fact that no signaling pathway operates in isolation, which necessitates further investigation into how this pathway interacts with other signaling mediators. Establishment and application of chemically defined culture conditions would greatly promote such investigations and diminish non-associated signaling interference. The nuclear factor-κB (NF-κB) and mitogen-activated protein kinase (MAPK) signaling pathways have been frequently reported to be implicated in disc degeneration [134–137]. Considering NF-κB and MAPK as downstream effectors of Akt, it is also important to clarify whether this pathway affects the development of IDD via regulation of NF-κB and MAPK. Hyperactivation of the PI3K/Akt pathway has been frequently reported in a wide variety of malignant tumors [138, 139]. Whether targeting this pathway in disc degeneration would lead to tumorigenesis is a considerable issue. Like MMPs, ADAMTSs are responsible for the degradation of Col II and aggrecan. It remains unclear whether this pathway inhibits ECM catabolism in the disc through downregulation of ADAMTS expression. In summary, more research into this pathway contribute to developing novel biological treatments for disc degeneration.
